# Parental sexual health development support for emerging adolescents in western upstate New York: a qualitative interpretive description study

**DOI:** 10.3389/fpubh.2026.1906863

**Published:** 2026-09-02

**Authors:** Natalie M. Leblanc, Sadandaula R. Muheriwa-Matemba, Aaliyah Gray, Osman Wumpini Shamrock, Joyonna Gamble-George, Mary Antwi, Danielle C. Alcena-Stiner, Dalmacio Dennis Flores

**Affiliations:** 1 University of Rochester School of Nursing, Rochester, NY, United States; 2 Center for Interdisciplinary Research on AIDS, Yale University, New Haven, CT, United States; 3 Behavioral, Sexual, and Global Health Lab, University of Rochester School of Nursing, Rochester, NY, United States; 4 University of Illinois-Chicago School of Nursing, Chicago, IL, United States; 5 Center for Women's and Gender Studies, Florida International University, Miami, FL, United States; 6 Center for Drug Use and HIV Research, New York University School of Global Public Health, New York, NY, United States; 7 Department of Epidemiology, Harvard T.H. Chan School of Public Health, Boston, MA, United States; 8 University of Pennsylvania School of Nursing, Philadelphia, PA, United States

**Keywords:** adolescent sexual health, anticipatory guidance, emerging adolescent, parent-child communication, Prepubescence, qualitative research, sexual health development

## Abstract

Parents and caregivers are critical in supporting emerging adolescents as they begin developing the foundations of sexual health and wellbeing. This study aimed to explore how parents and caregivers support sexual health development among emerging adolescents aged 10–14 years in Monroe County, New York. A qualitative interpretive description and conventional content analysis approach guided data collection and analysis. Purposive maximum variation and snowball sampling resulted in 20 participants being recruited and engaged in semi-structured in-depth interviews. Parents described sexual health development support as embedded within broader efforts to cultivate self-actualization, autonomy, emotional regulation, bodily awareness, hygiene, consent, and healthy relationships. Four themes characterized parental experiences: parental characterization of their influence on their child's personhood; parental support toward self-actualization; parental approaches toward sexual health development; and perceived threats to sexual health development. Parents reported using anticipatory guidance, open communication, boundary setting, and quality time to prepare their children for emerging social and emotional changes. Their approaches were shaped by their own childhood experiences, including trauma, neglect, cultural values, and prior gaps in sexual health communication. Parents also identified social media, peer influence, structural inequities, and community conditions as threats to healthy sexual development. Findings suggest that sexual health promotion for emerging adolescents should recognize parents as central supports motivated by trauma aversion and enacted through anticipatory guidance shaped by lived experiences, cultural values, and new threats such as social media. Findings conceptualize the parental role in sexual health development as part of whole-person self-actualization. Insights inform the design of trauma-informed, culturally responsive interventions.

## 1 Introduction

Early sexual debut (age first sexual encounter before or at age 14) is associated with increased susceptibility for lifelong adverse sexual health outcomes (1, 2). This timing of sexual initiation increases the likelihood of teen pregnancy, condomless sex with multiple partners, recurrent and delayed diagnosis of sexually transmitted infections (STIs) including human immunodeficiency virus (HIV), and unintended pregnancies in early adulthood (3–6). Currently, nearly 8% of young adolescents ages 10–14 in the United States report having sex, with twice the rates among males (10%) (7). In the 2023 National Youth Risk Behavioral Survey (YRBS), respondents who had reported sex before age 13 were less likely to use condoms during their last sexual contact than those who delayed sexual debut (74.7 vs. 85.4%) (8).

Recent surveillance data indicate that in Monroe County, New York, STI diagnoses increased by 16% between 2019 and 2020 among 15–19 year olds (9), and new HIV diagnoses increased by 26% between 2020 and 2021, among those younger than 25 years old (10). A 2023 study among adolescents aged 13–18 years found that the city of Rochester, located in Monroe County, had one of the highest rates of early sexual initiation, with about one-third of adolescents reporting sexual activity before age 15 (11). This rate was proportionally higher than the county average of 19% (12). Among those adolescents who report ever having sex in Rochester, 3% report having first sex before 13 years old, 4% had four or more sexual partners in their lifetime and 17% were sexually active (11). 43 percent (43%) of females reported that their male partners who used condoms and 54% of males in Rochester reported using condoms during their last sexual encounter (11); however, only 6% of sexually active females report using long acting reversible contraception and whose sex partner used condoms during the last time they had sex (11).

Early or emerging adolescence (ages 10–14 years) is marked by dynamic physical, mental, emotional, and social changes (13). Puberty, the biological and metabolic process of sexual maturity, typically commences between the ages of 10 and 14 in females and between the ages of 12 and 16 in males (13). This period is marked by the emerging adolescent's increasing desire for autonomy that is driven by neurological changes in brain development that heightened and imbalance between reward seeking and fluctuating self-regulation attributable to pubertal maturation (14). It is also at this juncture whereby preadolescent experiences and emerging curiosities may involve heightened susceptibility to adverse sexual health behaviors and related outcomes.

Brain regions associated with reward-seeking are highly sensitive to hormonal changes during puberty and may contribute to increased sensation-seeking and experimentation with oneself and the environment (15, 16). Therefore, the guidance and support young adolescents receive during this critical developmental period are essential for healthy sexual development. Inadequate support during this developmental stage is reported to have adverse physical, social and psychological outcomes, including poor mental health, emotional distress, social isolation, and engagement in health risk behaviors (17, 18). Outcomes related to such behavior like early pregnancy and recurrent STIs carry significant health, social, and economic costs. Unplanned pregnancy and STIs acquisition in early adolescence are associated with poor mental health, and adverse pregnancy and birth outcomes (19). Infants born to adolescent parents are more likely to experience low birth weight and higher rates of morbidity and mortality (20). Teen parents are more likely to leave high school prematurely, and experience long-term socioeconomic challenges that may affect autonomy and financial stability in adulthood. Early sexual debut and STIs among adolescents and young adult account for 60% of the $16 billion STI treatment costs (21, 22). Early sexual debut is also associated with lower feelings of self-worth and reduced ability to articulate boundaries or engage in interpersonal decision-making that requires negotiation (i.e., condom use, delayed sexual debut) (1, 2, 23). Additionally, early sexual debut is associated with low self-control in young adulthood, as well as decreased likelihood of pursuing educational or life course opportunities in adulthood, which has economic implications (24).

The World Health Organization defines sexual health as a state of physical, emotional, mental, and social wellbeing in relation to sexuality, and not merely the absence of disease, dysfunction, or infirmity (25). Consistent with this multidimensional definition, the concept of sexual health has evolved over the past few decades from exclusively a disease prevention and infirmary focused phenomenon to be more inclusive of disease treatment and care, pain-free, mental wellness, body autonomy, consent and most recently, pleasure. Consideration, however, is warranted to how sexual health is achieved across the life course, how young people are introduced to the topic, and experiences they have early on to ensure healthy sexual development. Development of sexual health and wellness typically begins early in one's life as part of one's self-actualization and developing personhood (25–28). Parental influence and familial values, peer norms and community contexts all influence the establishment of one's overall sexual wellbeing. How this is enacted early in one's life has also changed over time, where parents/caregivers and school-based expectations take on a larger role in supporting sexual health promotion and development early in the life course.

In this study, anticipatory guidance refers to parents' pre-emptive actions or responses that prepare their children for puberty, bodily changes, emerging curiosities, and sexual health development. Parents and guardians are best positioned to support the experience of their children as they navigate from prepubescent to young adolescence. During this process of sexual health development, parents and guardians are the preferred source of information by adolescents (29–32). Parents are a primary source of sexual health development for their children, because they provide messaging on family values and expectations about sex and promoting acceptable and appropriate science-based sexual behaviors for their children throughout adolescence (33). Additionally, parents are the most influential individual(s) in their children's lives at this time, and can provide sexual health information about sexuality, build upon ongoing interactions as children develop, and address misinformation learned from other sources (31). This role is especially critical during the period leading up to and including puberty, which is characterized by significant physiological, psychosocial, and brain development changes (33, 34).

Emerging adolescents rely on their parents to guide sexual wellness, and to initiate conversations based in part on their parents' lived experiences, information about sex, and reassurance about changes to their bodies and relationships (34, 35). Studies show that young adolescents are ready for sexual health information and acknowledge that it would be easier to delay sex and prevent pregnancy, HIV and other STIs if they were supported and had more open and honest conversations about sexuality particularly with their parents (32). However, these studies have not examined how parents conceptualize and practice anticipatory guidance during the prepubescent period, nor how their own childhood experiences, trauma, and cultural contexts inform these efforts-a gap this study addresses. Parent interactions with their children about sexual health and sexual development can support emerging adolescents in adopting healthy practices that minimize the potential for engagement in unhealthy sex-based behaviors and mitigate adverse sexual health outcomes (34, 36).

Communication that allows children to ask questions and receive honest responses has significant implications for adolescent sexual behavior. Direct, open, and responsive parent-child conversations about sex and parent monitoring of their child's sexual behavior have been associated with intentions to delay sexual activity, delayed sexual initiation, fewer sexual partners, condom negotiation and use, less influence from peers, and more accurate sexual health information among adolescents (33, 35, 37, 38). Studies show that parental support during this time delays sexual debut, and facilitates safer sex practices when they become sexually active in late adolescence and young adulthood (31, 39). Teens who perceive their parents as supportive and involved, and who are more satisfied with their relationships with their parents, tend to engage in less sexual risk behaviors (40). Despite the significant role and impact parents have in the sexual health development of their children, studies have shown that parents report discomfort, limited knowledge, and insufficient confidence when discussing HIV, STIs, and sexuality with their children, often citing embarrassment, fear of encouraging sexual activity, and lack of skills (41). When communication does occur, it's typically delayed, as many parents may underestimate adolescents' sexual behaviors and exposures, missing the critical window before sexual encounters begin (42). These barriers stem from intergenerational silence, lack of training or support, and societal stigma (43). Moreover, the parenting landscape has fundamentally shifted. Modernization, globalization, and digital access have expanded prepubescent and young adolescents' exposure to sex-themed content, while evolving norms around gender diversity and online sexual content require updated parenting strategies that caregivers may need assistance navigating. While some parents may lack comfort and communication skills, some are able to engage their children in sexual health conversations. However, a critical gap remains. Existing research has focused on parent-child sexual health communication during mid-to-late adolescence or after sexual activity has begun (7, 44). Comparatively little is known about how parents and caregivers support sexual health development during emerging adolescence (ages 10–14), a distinct developmental period when children are forming foundational knowledge, attitudes and skills regarding relationships, bodies, sex and gender, sexuality, and personal boundaries, but have not yet initiated sexual behaviors (45). Specifically, we lack understanding of how parents of early adolescents operationalize sexual health development guidance in practice, including what strategies they employ, what challenges they encounter in the digital age, and what resources including emerging online tools (46) they access and utilize to navigate these conversations. This gap is particularly pronounced in geographic contexts with elevated sexual health risks, such as Rochester, New York, where rates of HIV infection and other STI rates including congenital syphilis (47), and teen pregnancy, are highest than any other city in New York State outside New York City (48), and childhood poverty (49) create compounded vulnerabilities which are indicative of broader social and structural contextual problems. Within this context social and structural determinants of sexual health are exacerbated, rendering parental influence essential for mitigating heightened susceptibility to adverse sexual health outcomes. Yet, no research has examined how parents in this type of setting support sexual health development before behaviors that increase susceptibility emerge. However, no prior research has examined how parents navigate this 'new ecosystem' of social media and contentious discourse while supporting prepubescent children's sexual health development, nor how their own trauma histories and cultural contexts inform these practices. By examining parental perspectives, motivations and practices during this understudied developmental period and in this high-need geographic context, we aim to fill that gap and illuminate the strategies, challenges, and resources that shape early sexual health socialization in contemporary families.

## 2 Methodology

### 2.1 Study design

We used a qualitative interpretive description study design (3) as part of a community-based participatory research project (4, 5). Before conducting interviews, we held listening sessions with parents of children aged 10–14 years to help identify developmentally appropriate questions regarding parental experiences and perspectives supporting young adolescents' sexual health development. We selected a qualitative interpretive description approach (3) because very little is known about how parents support sexual health development among emerging adolescents, warranting a naturalistic description and interpretation of these experiences. Despite evidence that parent-child communication influences adolescent sexual behavior, little is known about how parents conceptualize and enact sexual health support during prepubescence (ages 10–14), a critical developmental window marked by the onset of puberty and heightened susceptibility to risk. Existing research has focused primarily on older adolescents and has not examined how parents‘ own childhood experiences, trauma, and cultural values shape anticipatory guidance during this earlier period. This study addresses that gap by exploring parental perspectives and practices in a high-STI/HIV-burden urban context.

### 2.2 Sampling procedure

Maximum variation and snowball sampling methods (6–8) were used to invite parents with specific criteria of interest. The principal investigators and study staff recruitment efforts sought participants from varied socio-economic status', age, and grade of the parent's young adolescent, to understand how sexual health development support is perceived, practiced, and understood. This approach helped to guide recruitment efforts to engage a diverse group of participants relevant to understanding sexual health support for young adolescents (8).

### 2.3 Recruitment

Active and passive recruitment strategies were used to engage parents. We circulated study flyers via email, posted flyers in healthcare facilities and leveraged relationships with community organizations. Subjects were asked to contact the study team directly and complete a screener form in REDCap to assess their eligibility. Eligibility included being over 18 years old, fluency in English, having a child aged 10–14 years enrolled in school in Monroe County, NY, and identifying as the child's primary caregiver, defined as living with the child at least 50% of the time. Participant screening was conducted by study staff either by phone, Zoom, in-person, and via an online screening survey created in REDCap. We obtained a waiver of written consent and all eligible participants received an informational sheet outlining the study details. After reviewing the information sheet, participants were prompted to indicate their interest by selecting either a “yes” or “no” checkbox to participate. If they selected “yes,” an interview was scheduled. Interested participants also provided their sociodemographic and contact information and indicated their preferred interview format (i.e., Zoom, in person or by phone), along with their availability for scheduling an interview.

The qualitative interpretive description research design allowed investigators to analyze and interpret the data while remaining close to participants' words and experiences (3). Thus, the interpretation allows the data to reflect participants' views, and the resulting data were detailed, plentiful, nuanced and meaningful. Studies show that this approach helps to accurately and systematically describe a population, situation, or phenomenon and facilitates a deeper understanding of the phenomenon of interest (3, 13).

### 2.4 Data collection

The interviews were conducted via Zoom or in-person, based on participant's preference, and lasted 35–65 min. Each participant received 30 USD for their time. A semi-structured in-depth interview guide asked questions within the following domains: (1) caring for a prepubescent child; (2) setting expectations; (3) sexual health support for young adolescents; and (4) conversations with your child about the changes you have seen within your child. The interview guide was curated by co-authors NML and SRM, and pilot tested by SRM with two participants. A debriefing session followed each interview to identify any unclear or confusing questions, which were subsequently revised. These pilot interviews were excluded from the main analysis. To maintain consistency, and as part of their post-doc training, co-author SRM conducted all the interviews. All data were collected in a manner intended to preserve participants' natural responses and experiences (3). Interviews were conducted from April to June 2023. The University of Rochester IRB provided regulatory approval.

### 2.5 Data analysis

A qualitative conventional content analysis approach was used to inductively, and iteratively generate codes, categories, themes, and sub-themes (9). Transcripts were generated and cross-checked for accuracy by co-authors SRM and NML. Co-authors SRM, AG and NML each read all transcripts repeatedly for data immersion while identifying the underlying concepts and clusters of concepts (9, 10). The co-authors (NML, SRM) are nurse research scientists and (AG) a behavioral researcher who engaged in reflexive memoing with each interview and discussed these memos during debriefing sessions to ensure interpretations remained grounded in participants' perspectives. Memoing deepened analysis in generating new ideas, and assisted in facilitating movement from raw data to broader conceptual abstractions that explained perspectives on sexual health (12). The initial codebook was developed in an Excel file and analysis was facilitated using Dedoose software. Coding continued systematically across the entire data set, with words, lines, and entire passages either forming a new code or merging an existing code (9–11). After developing the initial codebook, peer debriefings contributed to development and refinement of the final codebook a process that helped identify important interpretations and subsequent theme development (8). Data saturation and representation were assessed iteratively by the analysis team (NML, SRM, AG) after every five interviews; recruitment ceased when no new codes or themes emerged indicating that the data were sufficient to address the research question.

## 3 Results

All 20 participants were interviewed from January to June 2023 and primarily resided in the same jurisdiction. Most participants (*N* = 14) were mainly maternal, either mothers, caregivers or grandmothers, with the remaining participants (*N* = 6) being male, and were Black identified (*N* = 17). Participants ranged from 30–56 years old, were primarily Christian (*N* = 16), married or living together with a co-parent (*N* = 10) and reported completing a college degree (*N* = 15).

### 3.1 Theme 1: parental characterization of their influence on their child's personhood

#### 3.1.1 Sub-theme: parental personal upbringing

Parents were asked to reflect on their own prepubescent childhood memories. These memories provided insights into aspects of their upbringing that may influence their approach to parenting or caregiving. They were generally cognizant of how their personal upbringing had bearing on their approach to setting boundaries and expectations for their household and their children. As one father reflected on how his own experiences shaped his parenting approach, “….Just my experiences of what I‘ve gone through. I don't want to see her go through the same troubles or the same things that I've gone through…I want her to have a better life than me….I'm trying to raise her up different, I'm trying to give her what she needs…I'm trying to teach her right from wrong.”. Parents were also cognizant as to how their childhood experiences influenced efforts to curate the experiences and exposures, they wanted their young person to have. These experiences also undergirded the advocacy some parents engaged in to ensure their child's moral wellbeing and positive development are intact as their child progressed as a prepubescent into puberty. One paternal parent contrasted their own upbringing with the opportunities available to children today and emphasized preparing children with practical life skills: “I never grew up with this luxury of having all this technology, access to all this stuff. So, I want to make sure my kids have the skill sets and the opportunity to be successful. So, I try not to push them. I try to provide them the right skill sets in a toolbox that they can deal with the upcoming real life”.

Parents described formative childhood and prepubescent experiences, including trauma, neglect, parental absence, and other significant life events that influenced how they approached parenting and sexual health development with their children. Some experiences were more contextual and may include acute or long-term community and family-based trauma, or physical or sexual violence. As one mother reflected:

“[...] when things were really wrong and went astray, there was a lot of trauma involved. I mean, trauma on all kinds of levels. To give a little more insight, you could say abuse, physical abuse, emotional, verbal, psychological [...] A lot of times the things I found out growing up with body changes or menstrual cycles, I had to find out the wrong way. My mother didn't teach me [...] you always swear to yourself, you'll never allow yourself to go through it again or your child [...] And that's how it affected how I'm raising my daughter”.

Certain childhood experiences included being institutionalized and teenage pregnancy. Some parents also reported nuanced and positive experiences, that include cross-cultural, immigration and migration experiences alongside supporting their child through puberty. For these parents, they aimed for their child(ren) to have experiences similar to their own. One first-generation mother described her experiences growing up and wanting to ensure her children were influenced by her culture: “I was born and raised in West Africa, Liberia [...] I want to make sure that I'm instilling that in my kids [...] Most of my culture is shared in my house because I'm a first generation, so I know how to prepare our own food and I know a lot about our culture that I can have them participate in, so that helps”. Overall, these experiences shaped the values, practices, and forms of support parents sought to uphold or strengthen within their own parenting approaches and their children's developmental experience.

#### 3.1.2 Sub-theme: a good parent-child relationship

Parents' reports of good communication and spending quality time with their children indicated what parents perceived to be a good parent-child relationship. Parents were encouraged to reflect on their own childhood and communication with their parents. Parents characterized their childhood communication with their own parents as non-existent, strained, or contextualized through gratitude for their upbringing (i.e., single-parent household). These experiences influenced their intentions and practices toward communicating with their child about challenging and complex topics, including sexual health and overall wellbeing. For example, one mother explained, “My mother was never really open with us, I felt like my mom kept a lot of things hidden or she didn't want us to know [...] With my daughter, I definitely baby her but I'm very open to letting her know how I'm feeling, what's going on with me [...] I love this experience as far as teaching her to be open, and being able to come tell me [...] I love the fact that we have an open relationship, like we can talk”.

Parents generally described their relationships with their children as healthy and “good” as well as evolving and changing. Parents described open, direct communication as an indicator of a good relationship with their children. They spoke of these moments of communication alongside curating quality time between themselves and their child(ren). Parents emphasized their children's willingness to disclose sensitive or meaningful experiences as evidence of trust and relational closeness, particularly when children sought parental support or guidance. These instances of communication were indications that should the time arise where their child needed to communicate anything sensitive or of significance, their child would divulge to allow the parent to support them. One grandmother caregiver noted the importance of having an open policy with their child:

“You really want to give them that open door policy. You want them to come to you first with every little thing that they are imagining because if they're not coming to you, they are absolutely going to find the answer. And you don't want them to find it for themselves, and you don't want their friends to tell them because you already know that's not going to necessarily meet your standard. So you want to be the first. You want them to always feel like that they can get a solid answer from you”.

It is this communication and quality time that also provided confidence that they as parents were providing the support they believed their child needed and required at this time in the child's life. Parents overall aimed for open communication as a way of strengthening the relationship with their child but also a way of establishing boundaries and instilling family expectations and values.

### 3.2 Theme 2: parental support toward self-actualization

#### 3.2.1 Sub-theme: trauma aversion and anticipatory guidance

Some participants spoke of childhood experiences that they perceived as regrettable or traumatic. Due to these experiences, parents were very intentional on how they reared their children. Rearing involved establishing boundaries and having explicit expectations for their children to support development through puberty. Trauma aversion among parents influenced anticipatory guidance in developing the sexual health and overall wellbeing of their young person toward self-actualization. For example, one mother expressed, “[...] she's about to be 11 years old, I knew what it was like to not have a childhood and have it robbed from you [...] So I taught my daughter to speak up where I was taught to be silent, where I was forced to clean. I let it be a option to her [...] So I think the changes just continue to help her form into who she wants to be vs. forcing her to be someone I want her to be, or to grow up faster than what she needs to”.

#### 3.2.2 Sub-theme: puberty preparation and developmental support

Parents described observing physical, emotional, and social changes as their children transitioned from prepubescence into puberty. In response, parents employed a range of strategies to prepare and support their children through these developmental changes, including discussions about bodily changes, hygiene, emotional regulation, and self-esteem. Parents also described responding to their children's questions and using developmentally appropriate approaches to normalize puberty and support their child's sense of self. One mother used movies as a conversation point to discuss topics with her son:

“I try to bring a little humor to it to try to just ease the discussion. So we watched a Disney movie… and there's a character … he's a robot nurse and he's talking to this kid and he's like, ‘I'm scanning you and I'm seeing that you are entering puberty, you will grow hair here, here, here and-' [...] I was like, “Do you know what that means? Then we giggled about it, so that was probably how I initially approached the subject. I'm like, ‘It's when your voice will get deeper and you'll get hair and things, and things will grow and you'll start to look more like daddy”.

Parents also reported an array of strategies for navigating their children's emerging gender expressions and identities, whether conforming gender norms or not, alongside pubertal development. As one mother discusses about her child:

“I think she sort of identifies as non-binary, or gender fluid maybe [...] because I've known for a long time that she doesn't identify as a girl, but she's very developed, and that I think causes a lot of distress [...] Struggling with having a period, struggling with using tampons, struggling with using pads, they're not comfortable [...] we're trying to find a binder for her that fits really well right now, because she's really uncomfortable with her body”.

Communication was central in this regard as parents described efforts to support their child's sexual development; they also sought to build trust with their child while also helping to establish a sense of autonomy for their child.

#### 3.2.3 Sub-theme: emotional regulation support

To support their children with self-regulation of new emotions, tempering mood changes, and coping with peer dynamics, anticipatory guidance also entailed parents engaging their children in specific activities. Parents spoke of strategies employed to support children experiencing emotional distress or to mitigate ongoing emotional challenges. Some emotional distress may manifest through shifting affect, mood variability, challenges within peer networks, or difficulty managing evolving emotional responses during puberty. For example, one father reported encouraging his child to lift weights and exercise as a way of managing anger and other emotions. The father in this case spoke to seeing elements of anger that were reminiscent of his childhood experience and was attempting to temper this in his son with regular physical exercise and active participation in faith practices. As he mentions in his interview, “I don't know if this by genetics or biological but he have a little anger issue that worries me, that concerns me [...] I just try to teach him, ‘God is truth, that you need an education as well if you don't want to be put last' [...] He like to express his self by push up jumping jacks and he like the kicks”. As impending menstruation-initiated conversations with girls hinges on the possibility of pregnancy, boys in some instances were seen to need more emotional or behavioral support. As stated by one father, “I think for females it's more protection […] if you do one mistake, you could get pregnant. You could be a student mom basically and may ruin all your career, right? Well, for males this has to be I think more discipline and anger control”.

Other parents spoke of engaging their children in sports or in faith-based spaces not just to keep their child occupied, but to ensure curation of a healthy individual effect and exposure to healthy interpersonal experiences. For example, one father spoke to how he engaged his daughters in physical activities and ensured they were active in their use of sports as an outlet and as an approach to building their self-confidence:

“Now she just got her period a couple of months ago, she just got started playing basketball [...] Especially now my daughter, she is moving into middle school next year, make sure she has the confidence [...] New system, new teachers, new friends, you know how all this stuff can create some pressure on teenagers”.

These activities also served to balance academic/school-based activities. Some parents also spoke to engaging with their child in certain activities to spend quality time, but also to engage in conversation and get a sense of what their child's emerging interests are. A grandmother stressed the importance of this:

“we have to be as active in, and participatory as we can with our kids [...] We really need to participate in what they like and what they're interested in.” For example, the mother of the nonbinary adolescent regularly watches YouTube with them: “she has a favorite YouTuber [...] every Friday [...] she waits for me to watch, because we watch together [...] but at least her coming back for a connection.”

A mother who experienced a pregnancy as a teenager described spending time reading about menstruation with her daughter, “I had gotten some books from the library, and we sat down, read them together, and had the whole discussion about her period and things like that. She was very into it, very interested”.

### 3.3 Theme 3: parental approaches toward sexual health development

#### 3.3.1 Sub-theme: trauma as leverage to inform approaches to sexual health

Trauma aversion together with anticipatory guidance informed parental efforts to support the sexual health development of their children undergoing the prepubescent – puberty process. Through reports of their childhood experiences, study participants did not express themselves being engaged in any type of comprehensive sexual health education or communication with their parents. As one grandmother caregiver noted, “I grew up in a strict Christian household, so they weren't telling us about rubbers and all of that. Only thing they told us is to keep your legs closed. Keep your dress down, keep your legs closed”. Therefore, parents' childhood experiences informed how they approached sexual health development with their own children or grandchildren, particularly through trauma aversion and anticipatory guidance. Trauma aversion and anticipatory guidance influenced the environments parents were willing to allow their children to engage in socially. These experiences also informed with whom and where parents and caregivers were comfortable to allow their children to receive sexual health and other sensitive information. As stated by one maternal caregiver, “I believe in educating my child at home” and as one mother noted, “I feel like I can give her more information vs. anybody else can give her, because I feel like I'm more educated than anybody else to give my kids advice about [sexual health]”. When asked if school-based sex education could support parents talking to their children about sexual health, another mother echoed these sentiments: “I don't know if [a school] can do better than their parents or their grandparents or whatever, but that's my thing”.

Trauma aversion motivated how parents approached sexual health communication and establishment of boundaries and expectations with their children. For parents, sexual health communication motivated by trauma aversion and anticipatory guidance was influenced by a desire to prevent negative experiences from their own childhood while drawing on lessons, values, and parenting knowledge acquired through lived experience or formal resources. This learning could have been from a more formal resource in tandem with their own lived experiences.

Parental anticipatory guidance was a main feature in parental efforts to support sexual health and wellness, and overall wellbeing for their prepubescent child. Parental anticipatory guidance refers to the parent's pre-emptive actions or responses in preparing their child for puberty. This guidance was either parent initiated and/or child initiated. Parent initiated guidance was in preparation for or in response to physiological changes children were undergoing or about to undergo given the child's biological age. For example, one mother reflected on her experience growing up: “I have had the conversations with them both about condoms and my daughter about birth control. I wish that I would've been on birth control like that. I could've came to my parents and told them that I wanted birth control, but I just felt as though I couldn't talk to them.”. On the other hand, another caregiver described her nephew approaching her for guidance: “He comes to us and he'd like, ”Girl who was [I] talking and you told me, wow, now what I'm supposed to do?”.

Parents' sexual health development support is geared first and foremost toward puberty preparation and guidance. Parents were preparing their prepubescent to bodily changes and mood and setting boundaries and expectations. These changes entailed anatomical and hormonal changes that influenced gender expression, hygiene, new body odors associated with adrenarche and shifts in mood and affect during puberty. Changes were also a result of increasing peer influence and the centering of peer relationships that impact their child's all-around personhood. Subsequently, the content of sexual health communication, and by extension sexual health development, was not primarily geared toward understanding the act of sex itself, but rather for their child to understand sex-based anatomy and processes, as well as strategies for emotional intelligence. Parent support also focused on promoting the child's autonomy in decision-making and expression, promoting self-hygiene, and ensuring there was an understanding of consent in peer relationships, all culminating in puberty preparation. As one grandmother noted, she wants to prepare her granddaughters to take control of their hygiene:

“Well, I expect even with caring for themselves daily, making sure that they don't have any odors. Taking care of themselves, being aware of their bodies. If things are changing, if something is happening that wasn't happening before, being able to say I need some help. This isn't normal. And getting the help that they need”.

#### 3.3.2 Sub-theme: sexual health development communication

While anticipatory guidance reflected broader preparatory parenting strategies and responses to developmental changes, sexual health development communication referred more specifically to the direct conversations parents had with their children regarding puberty, bodily changes, hygiene, relationships, peer interactions, and sexual health topics. These impending or actual changes were either anatomical in nature, responsive to emerging hygienic and grooming needs, or other evolving body processes. One mother reflected on this process with her son:

“[...] it started off with just a stronger body odor and then he started to grow some hairs on his back [...] then when he started to perspire, it was like, “Okay, I think it's done that we get to deodorant stage, because of course you‘re becoming a teenager and all of this hormonal changes is going to start happening.” That's when we started talking about, “Hey, you got to make sure you're showering and putting the other way on,” and stuff like that”.

These physical and social changes are compounded by parents' concerns regarding peer influence or observation of their child's experiences with peers. Communication was initiated as parents observed among their children, changes in peer relationship dynamics that in some cases caused emotional distress. As one mother noted about her son, “he's starting to hear things that different kids around the school will say, or gestures. Some of the kids are starting to make sexual type gestures, and I don't even know if they know what it means, but they‘re doing it, and then my kid will repeat it or tell me about it”. Similarly, one father described initiating a conversation with his daughter after she became concerned about a pregnant classmate. He explained that the conversation became an opportunity to reinforce prior discussions about sex, intimacy, and the potential consequences of sexual activity.

Anticipatory guidance initiated by the child or other children includes age-appropriate sexual health communication motivated by child or sibling curiosities. In these cases, children either pose questions about themselves or siblings experiencing physical changes or observing parental experiences that may be of a sexual or reproductive nature (i.e., pregnancy, menstruation). These conversations also entailed prepubescent inquiry based on observations within their peer groups, which warranted explanation or detailed discussion such as that mentioned by a father whose daughter's classmate became pregnant:

“She mentioned a 12-year-old girl in school was pregnant. She got pregnant for a 14-year-old eighth grader, and she's a seventh grader and is pregnant. So my daughter came home a little disturbed by that and concerned for the girl [...] we had to have conversations but we already had the conversation about what sex is. So it was just really a matter of reinforcing why it's important to not have those type of interactions or intimacy until you are a grown up and can be prepared for the potential consequences of having sex”.

#### 3.3.3 Sub-theme: sex and gendered dimensions of sexual health promotion

The influence of gender on prepubescent adolescent sexual health promotion emerged in two distinct ways. Sexual health promotion manifests through a sex-as-biology lens of puberty, whereby menstruation as a marker of pubertal development further characterized girls as “early” or “late bloomers” based on the start of menarche. Signs of puberty for sons were less clearly defined with parents mentioning physical and emotional changes. As several parents noted, daughters were more interested in discussing puberty as well, which reinforced parents' positioning girls as a primary focus for sexual health discussion within families. Additionally, girls' pubertal experiences may be juxtaposed to how young males are advised to assess their sexual health and how health promotion is even viewed. This is highlighted in one caregiver's statement that demonstrates how anticipatory guidance of her nephew is associated with a girls' pubertal development: “We started talking to him when he was about 12, and you start talking about condoms and protect yourself against certain young girls because it is not always boys that try and do things […] And it's not just look watching out for pregnancy. It's the diseases. And now diseases are out there that you cannot get rid of”.

Parents' emphasis on menstruation extends to concerns about unintended pregnancy and may have been biased toward girls. This further contributes to a gendered lens of puberty as highlighted by one mother's comment, “Boys don't get pregnant, so they don't have to worry about that. They don't have to worry about their periods. They basically just have to worry about wrapping it up. Girls have a lot more that they deal with than boys” and as a grandmother caregiver noted this gendered lens disallows for a balanced sexual health conversation: “I mean sometimes we make this a girl conversation, but it's a boy conversation as well too. They have to have conversations about what it looks like to be a healthy young man as well. And not putting that all on just the girls but the boys do too”.

Another way is how mothers/maternal influences on sexual health promotion compared to fathers/paternal influences on sexual health communication. Parents described gender preferences in that mothers acted as the primary facilitator of sexual health communication in the household. Some of the fathers interviewed underscored how critical mothers are in sexual health communication specifically. One father discussed the importance of his wife as the “go-to person” in sexual health communication for their daughters especially in instances where they may not be comfortable discussing puberty changes with him because they are “girl children”. Another father, who described his close relationship with his son, discussed his wife being the primary communicator for their son: “He won't come to me [...] I don't know what it is, but they always tend to go to their mother [...] He's always with me [but] They always go to their mother first”. Mothers were often described as the primary facilitators of sexual health communication, while fathers and other male caregivers more commonly reinforced broader relational and behavioral expectations. As noted by one mother:

“[...] I'm the primary educator around these topics between their dad and I […] I think my son's grandfather has talked to him a few times about things, just mainly around respect and what a healthy relationship looks like and things like that. But yeah, that's about it. I wouldn't say beyond that. It's primarily been me, I would say 90% and 9% their dad”.

Fathers' and male caregivers' sexual health communication with their sons often focused on establishing integrity, emotional and behavioral regulation, and navigating dating or relationships with the opposite sex, underscoring how mothers take on topics more specific to pubertal development. Mothers described preferences navigating sexual health conversations with their sons, expressing concerns that they do not have the knowledge or words to discuss the male experience that a father would have, as noted by one mother who mentioned she is procrastinating having these conversations with her son, and another mother who described her discomfort:

“I was more comfortable with my son not wanting to talk about it at the time just because, I really would rather his dad talk to him. But I'm the one that's home. His dad is at work. I think the next time we try with him, it'll be his dad having that conversation [...] My husband is oblivious to the world unless I tell him, ‘Listen, you need to sit down and talk about this.' He just doesn't recognize any issues”.

Lastly, anticipatory guidance was reported in communication about establishing boundaries and setting expectations for the household and subsequently for the participant's children.

### 3.4 Theme 4: threats to sexual health development

#### 3.4.1 Sub-theme: interpersonal engagements and influences

As parents reported efforts toward supporting their child's sexual health development and self-actualization, they also reflected on concerns and threats to the sexual health of their young person. Parents who were Black men or raising Black boys spoke on efforts to not only build resiliency and personhood for the child but also spoke of consistent advocacy for their child. These parents spoke about concerns of their child's well-being and societal threats in institutional and social settings which they have had to address. As one mother noted, “Looking at my 10 year old, being that he's a boy, I feel like some of the stereotypes that is out there regarding Black boys, for me that is very hard because I see him and I think, okay, he's going to have to go to do that. Not by choice, but he's just going to be another Black boy walking down the street and something's going to happen just because he is [Black]”. Another father spoke to their child entering school and potentially being exposed to other students who may have had certain experiences:

“Definitely when she goes to middle school we‘ll be worried. Because they go in school and she'll be the youngest in the school. She may have a ninth grader next to her and we all know the ninth graders are fully developed. Some of them, if not all of them already had a sexual experience in their life. So it's easier for them to put some peer pressure and everything.”

Parents pointed to external factors that they perceived as a threat to their child's personhood and subsequent healthy sexual development.

#### 3.4.2 Sub-theme: social media content and use

All parents described concerns regarding social media by reporting either specific content that was deemed inappropriate and intentionally monitoring their child's use of social media or regulating content exposure. This is due to some content on the surface appearing benign and age-appropriate, but upon further review in some cases parents were left concerned by the content. Some of this content was perceived to be overly violent or sexualized among familiar characters. One mother recalled an experience with her son: “I noticed that he Googled pregnancy or something like that and he found a cartoon in YouTube. They were not cartoons, they were people like dressed like Spider-Man and Spider Girl, and the Spider Girl was pregnant, and we went nuts”. As an extension of establishing expectation and boundaries, parents enforced limits on social media use, including both the platforms their children accessed and the content they viewed. Several parents also mentioned using software to block content and regulate their child's exposure as a way to curate a developmentally appropriate information environment.

## 4 Discussion

Despite evidence that parent-child communication influences adolescent sexual behavior, little is known about how parents conceptualize and enact sexual health support during prepubescence (ages 10–14), a critical developmental window marked by the onset of puberty and heightened susceptibility to lifetime adversity. The extant research focuses primarily on older adolescents and has not examined how parents' childhood experiences, trauma, and cultural values shape anticipatory guidance during this early transitionary period. The extant research also tends to use a deficit lens, which disallows adequate exploration of how parental strengths can be leveraged to align with evidence-based information. This study addresses that gap by exploring parental perspectives and practices in a high-STI/HIV-burden urban context.

Our findings demonstrate that emerging adolescent sexual health development support is embedded within parental efforts toward curating self-actualization for their young person growing through physical and emotional maturation. Within these efforts, parents actively employed anticipatory guidance, both self-initiated and in response to their children's curiosities, to prepare their prepubescent children for the onset of puberty and emerging adolescence. This guidance included discussion of bodily changes, emerging hygiene needs, cultivating emotional intelligence, building autonomy, and understanding consent within peer relationships. This approach is crucial, as the emerging adolescent period (ages 10–14 years) is a time of dynamic physical, mental, emotional, and social changes, making parental guidance and support essential for healthy sexual development (13, 33). Prior research has documented parent-child communication about sex in older adolescents, but this study is the first to show that parental support during prepubescence is conceptualized as part of whole-person self-actualization, encompassing hygiene, emotional regulation, autonomy, and consent-not just sexual behavior. Parental approaches reported in this study align with the evolving understanding that sexual health is a part of whole-person wellness across the life course, and parents or caregivers are instrumental for setting the course of a healthy sexual life. This developmental process begins in pre-adolescence (25, 27, 28, 50), signifying the importance of this time and parental influence when considering one's life course. Overall, parental efforts were pre-emptive to avoid adverse experiences and leverage assets from learned lessons to set boundaries and expectations with their child(ren).

Parental childhood experiences further informed a parenting approach of anticipatory guidance in shaping the sexual health development experience of their young person. Findings revealed that sexual health development support provided through anticipatory guidance was informed by parents/caregivers' own childhood experiences. Some participants reported personal experiences of sexual and social traumas, such as molestation, teenage pregnancy or community violence. Others reported emotional neglect or certain contexts that made growing up challenging or reflecting on how they desire to parent. Parents also reported experiences, values, and practices they hoped to retain for their children and uphold, expound upon or were positively memorable and impactful. Parents reflected on their own childhoods, where communication with their parents about sensitive topics was either non-existent, strained or outcome-centered (i.e., “keep your legs closed”). Therefore, parental efforts are intentional. While prior research has examined parents' communication strategies, this study is the first to document trauma aversion as a central motivator of anticipatory guidance during a child's emerging adolescence, and to show how parents' own childhood experiences-including trauma, neglect, and cultural values-shape their approach to supporting their children's sexual health development. Parents' communication and enforcement of boundaries and desires for their children did not occur in a vacuum, but informed by these lived experiences within a specific context. These experiences profoundly influenced their intentional efforts to foster open dialogue with their own children, particularly regarding complex subjects like sexual health and overall wellbeing.

Parents expressed a desire to be the primary source of information for their children, believing they were best positioned to provide accurate and value-aligned guidance. Parents utilize a variety of strategies to initiate and sustain these conversations. They observe their children's physical and emotional changes, such as new body odors or mood shifts, and use these as natural entry points for discussions about hygiene and hormonal changes. Humor, as demonstrated by one parent to discuss puberty, can ease discomfort and make these conversations more accessible. Parents also adapt their guidance to address emerging gender expressions, supporting children who may be experiencing distress related to their developing bodies. Beyond physical changes, anticipatory guidance also involves supporting children with emotional regulation, helping them navigate new emotions, temper mood changes, and cope with peer dynamics. This might include encouraging participation in sports, faith-based activities, or simply engaging in shared interests to foster connection and provide outlets for emotional expression (71–75).

Findings also suggest that parents may need support in providing sexual health development support for their young person. Therefore, interventions that seek to shape adolescent and pre-adolescent sexual health should be cognizant that parents are generally the best person to engage young people, or other extended family members serving caregiving roles, such as aunts, uncles and other caretakers. Research demonstrates that young people prefer parental influence in their lives with regards to their sexual health and sexuality (29–32). Intervention development should be mindful and attend to the fact that different cultural and religious values do not necessarily need to be in opposition to sexual health promotion but can embody them (51). Community-informed interventions would ensure that these values and expectations rooted in culture and history can be leveraged and aligned with evidence-based strategies to promote sexual health for young people. Therefore, there is a critical need to examine the experiences during early adolescence that support young adolescents' sexual health development.

Employment of anticipatory guidance in the context of sexual health development among participants in this study, was informed by personal trauma experiences, and motivated to avert these experiences for their child. Additionally, there is a desire to build autonomy and wellbeing. Based on our findings that parents' approaches are shaped by trauma, cultural values, and lived experiences, we recommend that comprehensive sexual health education programs consider these contexts when designing curricula. Comprehensive sexual health education from preadolescence to adolescence should intentionally consider historical, intergenerational, familial, and cultural contexts. Doing so may help balance evidence-based sexual health information regarding relationships, physical and mental hygiene, STI prevention, and unintended pregnancies with community values and lived experiences. The effort toward balancing cultural strengths and sexual health and wellness must give grace to communities with compounding determinants and work toward culturally tailored interventions. These cultural elements can be leveraged to strengthen whole-person wellbeing. However, further research is warranted to evaluate whether such trauma-informed tailoring improves parent-child communication or adolescent outcomes.

Parental efforts seeking to balance science, lived realities, and engender autonomy in supporting sexual health development during this time is crucial more than previous generations. Parenting and support for sexual health development has now evolved to monitor new and old external factors such as peers and the interpersonal environment that have an influence on one's personhood. For the first time in human history parents are having to contend with an ominous presence that has profound influence on their children's lives and the lives of their peers – technology and social media. Findings in the current study described parents vigilance regarding social media and technology exposure to their young person. The use of social media and technology, and now the emergence of artificial intelligence (AI) have made parents wary of this influence in their child's lives (52). It is a newer external factor that has had an impact on overall wellbeing and the sexual health of young people. Research and public discourse have documented threats to adolescent wellbeing and sexual health as result of adolescent engagement in the emerging digital spaces. Reports of AI – enabled suicidality and increasing sexual extortion experienced by prepubescents and adolescents is a real concern (53–60). Thus requiring parents to now incorporate curating discernment regarding use of tech and exposure to social media as part of sexual health development and communication with their children. Parents could use support as they navigate this new socio-ecological terrain.

Despite variation in community values and household expectations among participants, findings suggest there are certain sexual health tenants that are cross cutting at young ages, transcend cultural nuances, and expound beyond one's sexual orientation, gender expression and sexual behavior. Our findings suggest that certain sexual health concepts-such as anatomy, sex at birth, gender, hygiene, body autonomy, and consent is foundational across diverse contexts. Additionally, it is the knowledge of basic human physiology and processes (i.e., menstruation, hair growth and voice changes) and body functions in tandem with self-preservation, self-actualization, mood regulation and hygiene related to the physical body. The understanding with the vocalization of appropriate language for oneself and respect for oneself vis-a-vis others we posture all cross-cutting concepts that are important in the sexual health development of young people. This is regardless of who future sex partners will be and what orifice and body parts are used in sexual activities. Equally important is the understanding that seeking intimacy is a normative human experience (25, 26, 33, 61), and that there are ways of intimacy that do not have to involve penetrative behavior and can be posed as an option to delay sexual debut in adolescence (33, 39). It is crucial that young people have a firm understanding of these concepts. However, our sample was drawn from a single geographic area, and further research is needed to examine the universality of these concepts and how they are interpreted and enacted differently across cultural contexts.

Parents are often the primary sources of information for their children. Therefore, efforts should revolve around strengthening parent's capacity, confidence, and knowledge regarding sexual health development for their young person. It is important for stakeholders and educators in the sexual health space to understand that, generally, parents operate from a place of trauma aversion and legacy building. Although there may be exceptions to this, it is important for stakeholders to attempt to align with parental desires and seek a point of confluence. At times there may appear to be discord between household and family values and beliefs vs. perceived mainstream sexual health norms and discourse. However, exploring undergirding alignment can assist with designing approaches that include parents and other family systems to tailor messaging and guidance for parents and sexual health promotion efforts. This is particularly essential as ubiquitous presence of social media and large language models with AI are becoming prominent in the lives of young people. It is becoming increasingly important that certain fundamentals are understood and remain central as definitions of sexual health continue to expand beyond disease status and prevention, and include consent, respect, and pleasure.

Development of sexual health and wellbeing typically begins early in one's life as part of self-actualization. In this study, parental influence and familial values, peer norms and community contexts all impact on the establishment of one's overall sexual wellbeing. For some, sexual health problems in adulthood were influenced by familial and peer trauma or harm embedded within adverse community and historical contexts. For others, sexual wellbeing is carefully directed by parental and familial guidance despite adverse community contexts. For many, however, the development of sexual health wellness and wellbeing are pursued with intention during late adolescent into adulthood. This is in part because the focus of public discourse regarding sexual health, specifically HIV/STIs transmission, tends to be concentrated among older adolescents and adults (62, 63). Intervention development must include an understanding that the parental approach to sexual health development is motivated by anticipated experiences specific to their environment and may be motivated by parent's childhood traumas and lived experiences. Our findings that parents operate from a place of trauma aversion and legacy building suggest that interventions may benefit from aligning with parental values and seeking points of confluence. However, further research is needed to evaluate how alignment can occur and what is required to improve parent-child communication, adolescent outcomes, or program uptake.

Parents' narratives provided the rationale and requirement for sexual health promotion, education and intervention to be trauma-informed, culturally and contextually centered (51, 64–66). Additionally, the local setting of this study is known for its abject child poverty, a context whereby adverse community-based experiences such as fatal community violence are heightened. Research has shown that community distress can influence adverse sexual health outcomes, like heightened susceptibility to teen pregnancy, congenital syphilis and HIV/STI acquisition (67–70). Compounding this reality is when other community systems are perceived as threatening their child's sexual self-actualization and wellbeing, parents may harbor distrust for such systems. For example, children having to confront school-based discrimination based on their race and gender are experiencing threats to their wellbeing, yet parents are asked to trust these same spaces to support their children's sexual health development. The educational environment may now be perceived as a contentious space that may disallow any type of strength-based support for children. Therefore, parents may attempt to avoid generational adversity, while leveraging generational and cultural knowledge with new evidence. School or policy-based interventions that do not consider this context can be perceived as being disruptive and antagonistic to the parental role, as well as dismissive to cultural or historical realities that are perceived by communities to be protective. This may be perceived as a personal threat to parents and their efforts to build personhood among their children, subsequently engendering community distrust. At the same time, efforts to engage parents in sexual health promotion should not inadvertently restrict adolescents' access to medically accurate, developmentally appropriate sexual health education. Prior scholarship has noted increasing tensions between parental authority, adolescent autonomy, and access to evidence-based sexual health information, particularly within school and community settings (63). Therefore, community-informed interventions may benefit from positioning parents as collaborative partners while also centering adolescents' rights to accurate health information and their preferences for trusted professional health educators.

Parents are navigating new social and technological terrain which has proven challenging, while continuing to support their children as they embark through adolescence. Providing parents with support is both an opportunity to leverage and expand access to sound sexual health promotion for emerging adolescents.

## 5 Limitations

A strength of this study is the use of a qualitative descriptive-interpretive approach to center parents' and caregivers' accounts of supporting sexual health development among emerging adolescents. Despite the significance of the findings, there are some limitations. Participants were recruited from Monroe County, New York, and findings may not fully reflect the experiences of parents and caregivers in other geographic or sociocultural contexts. However, the aim of qualitative research is for contextualization of a phenomenon and lived experiences that may have merit in other settings. Participants were recruited from Monroe County, NY, a high-burden small urban center with elevated rates of childhood poverty, HIV/STIs. This context may have heightened parents' vigilance regarding threats to their children's sexual health (e.g., social media, peer influence, community violence), and findings may not generalize to lower-burden or rural contexts where structural determinants differ. Future research should examine parental experiences across diverse geographic and socioeconomic settings. The study also included English-speaking parents who self-selected into the study, which may have resulted in a sample of caregivers who were more comfortable discussing parenting, puberty, and sexual health. Self-selection may have led to overrepresentation of parents who are motivated to prevent trauma for their children or who have strong opinions about school-based sex education. As a result, the findings may not capture the experiences of parents who are less engaged, less comfortable, or less resourced to support their children's sexual health development. Additionally, although interviews were conducted in private settings and emphasized confidentiality, social desirability may have influenced how participants described their parenting practices. Social desirability may have led to overreporting of open communication and underreporting of punitive approaches or avoidance of certain sexual health topics. Likewise, a sustained deficit lens and approach in research may have influenced participants overreporting reports of challenges and needs. Future research can include a broader range of caregivers, including non-English-speaking families, parents from different geographic contexts, and young adolescents themselves, to further contextualize sexual health development support within families.

## 6 Conclusion

Despite potential limitations, the research design and approach sought rigor in illuminating participant experiences and discussion of implication for research, and practice. Parental support for sexual health development among emerging adolescents is embedded within efforts to cultivate self-actualization, bodily awareness, emotional wellbeing, autonomy, and healthy relationships. Parents in this study described anticipatory guidance as an intentional practice shaped by lived experiences, trauma aversion, cultural values, family expectations, and community realities. These efforts were not limited to conversations about sex, but included preparing young adolescents for puberty, hygiene, consent, peer dynamics, gendered expectations, emotional regulation, and social media exposure. Findings suggest that sexual health promotion for young adolescents should attend to the needs of parents and caregivers as they navigate this developmental period with their children. Family-centered, trauma-informed, and culturally responsive approaches may help strengthen parent-child communication while also supporting young people's access to accurate, developmentally appropriate sexual health information. Centering parents as the primary and initial stewards and subsequent partners in adolescent sexual health promotion offers an opportunity to leverage existing family strengths while advancing young people's autonomy, safety, and overall well-being.

## Data Availability

The datasets presented in this article are not readily available because raw qualitative interview transcripts cannot be publicly shared due to assurances of confidentiality provided to our participants as per the informed consent agreement, and the possibility of individual identification based on information revealed in the interviews. Requests to access the datasets should be directed to natalie_leblanc@urmc.rochester.edu.

## References

[1] Golden RL Furman W Collibee C. The risks and rewards of sexual debut. Dev Psychol. (2016) 52:1913–25. doi: 10.1037/dev0000206 27709996 PMC5117815

[2] Kaestle CE Halpern CT Miller WC Ford CA. Young age at first sexual intercourse and sexually transmitted infections in adolescents and young adults. Am J Epidemiol. (2005) 161:774–80. doi: 10.1093/aje/kwi095 15800270

[3] Centers for Disease Control and Prevention. HPV vaccine for preteens and teens Atlanta, GA: CDC; 2021 [cited 2021]. Available online at: http://www.cdc.gov/vaccines/parents/diseases/hpv.html (Accessed March 1, 2024).

[4] Epstein M Bailey JA Manhart LE Hill KG Hawkins JD Haggerty KP . Understanding the link between early sexual initiation and later sexually transmitted infection: Test and replication in two longitudinal studies. J Adolesc Health. (2014) 54:435–41. doi: 10.1016/j.jadohealth.2013.09.016 24280303 PMC3965628

[5] Magnusson BM Masho SW Lapane KL. Early age at first intercourse and subsequent gaps in contraceptive use. J Womens Health. (2012) 21:73–9. doi: 10.1089/jwh.2011.2893 PMC3283439 21992618

[6] Pingali C Yankey D Elm-Evans LD Markowitz LE Williams CL Fredua BF . National, regional, state and selected local area vaccination coverage among adolescents aged 13–17 years in the United States, 2020. Morbid Mortal Wkly Rep. (2021) 70:1183–90. doi: 10.15585/mmwr.mm7035a1 34473682 PMC8422873

[7] Muheriwa-Matemba SR Anson E McGregor H Zhang C Crooks N LeBlanc NM. Prevalence of early sexual debut among young adolescents in ten states of the United States. Adolescents. (2024) 4:440–52. doi: 10.3390/adolescents4030031

[8] Szucs LE Pampati S Jozkowski KN DeGue S Rasberry CN Brittain AW . Asking for verbal sexual consent and experiences of sexual violence and sexual behaviors among high school students: youth risk behavior survey, United States, 2023. MMWR. (2024) 73:59–68. doi: 10.15585/mmwr.su7304a7 39378231 PMC11559680

[9] Valenti WM. Sexual health in Rochester/Monroe County: Sexually transmitted infections are a shared problem with shared solutions. Trillium Health. (2021).

[10] Hochul K Zucker HA Proud KM. Health advisory: Increased number of HIV diagnoses in Erie and Monroe counties. New York, NY: Department of Health. (2021).

[11] Bello A Vélez de Brown M. 2023–24 school year Rochester City School District youth risk behavior survey report. Rochester, NY: Monroe County Department of Public Health; 2024.

[12] Bello A Vélez de Brown M. 2023–24 school year Monroe County youth risk behavior survey report. Rochester, NY: Monroe County Department of Public Health; 2024.

[13] Greenberg JS Bruess CE Oswalt SB. Exploring The Dimensions Of Human Sexuality. 6th ed. Burlington, MA: Jones & Bartlett Learning; 2017. 824 p.

[14] Pfeifer JH Allen NB. The audacity of specificity: Moving adolescent developmental neuroscience towards more powerful scientific paradigms and translatable models. Dev Cogn Neurosci. (2016) 17:131–7. doi: 10.1016/j.dcn.2015.12.012 26754460 PMC4826651

[15] Kobak R Abbott C Zisk A Bounoua N. Adapting to the changing needs of adolescents: parenting practices and challenges to sensitive attunement. Curr Opin Psychol. (2017) 15:137–42. doi: 10.1016/j.copsyc.2017.02.018 28813254 PMC5886742

[16] Shulman EP Smith AR Silva K Icenogle G Duell N Chein J . The dual systems model: review, reappraisal, and reaffirmation. Dev Cog Neurosci. (2016) 17:103–17. doi: 10.1016/j.dcn.2015.12.010 26774291 PMC6990093

[17] Camara M Bacigalupe G Padilla P. The role of social support in adolescents: are you helping me or stressing me out? Int J Adolesc and Youth. (2017) 22:123–36. doi: 10.1080/02673843.2013.875480

[18] Kwon JA Wickrama KA. Linking family economic pressure and supportive parenting to adolescent health behaviors: two developmental pathways leading to health promoting and health risk behaviors. J Youth Adolesc. (2014) 43:1176–90. doi: 10.1007/s10964-013-0060-0 24254978

[19] Chesson HW Mayaud P Aral SO. Sexually transmitted infections: Impact and cost-effectiveness of prevention. In: Holmes KK, Bertozzi, S, Bloom, BR, editor Major infectious diseases 3rd ed Washington (DC): The International Bank for Reconstruction and Development / The World Bank. (2017). 30212101

[20] DeMarco N Twynstra J Ospina MB Darrington M Whippey C Seabrook JA. Prevalence of low birth weight, premature birth, and stillbirth among pregnant adolescents in canada: a systematic review and meta-analysis. Journal Pediatr Adolesc Gynecol. (2021) 34:530–7. doi: 10.1016/j.jpag.2021.03.003 33727190

[21] Centers for Disease Control and Prevention. Incidence, prevalence, and cost of sexually transmitted infections in the United States, 2018 Atlanta, GA: CDC; 2024. Available online at: https://www.cdc.gov/nchhstp-newsroom/factsheets/incidence-prevalence-cost-stis-in-us.html (Accessed March 1, 2024).

[22] Ventura SV Hamilton EB Mathews TJ. National and State patterns of teen births in the United States, 1940–2013. Hyattsville, MD: CDC. (2014). 25142408

[23] Magnusson BM Crandall A Evans K. Early sexual debut and risky sex in young adults: the role of low self-control. BMC Public Health. (2019) 19:1483. doi: 10.1186/s12889-019-7734-9 31703650 PMC6839049

[24] Parkes A Wight D Henderson M West P. Does early sexual debut reduce teenagers' participation in tertiary education? Evidence from the SHARE longitudinal study. J Adolesc. (2010) 33:741–54. doi: 10.1016/j.adolescence.2009.10.006 19897236 PMC2946557

[25] World Health Organization. Defining sexual health Geneva, Switzerland: World Health Organization; 2026 Available online at: https://www.who.int/teams/sexual-and-reproductive-health-and-research/key-areas-of-work/sexual-health/defining-sexual-health (Accessed February 1, 2026).

[26] Zaneva M Philpott A Singh A Larsson G Gonsalves L. What is the added value of incorporating pleasure in sexual health interventions? A systematic review and meta-analysis. PLOS ONE. (2022) 17:e0261034. doi: 10.1371/journal.pone.0261034 35148319 PMC8836333

[27] World Health Organization. Redefining sexual health for benefits throughout life: World Health Organization; 2022 Available online at: https://www.who.int/news/item/11-02-2022-redefining-sexual-health-for-benefits-throughout-life (Accessed March 1, 2024).

[28] Edwards WM Coleman E. Defining sexual health: a descriptive overview. Arch Sex Behav. (2004) 33:189–95. doi: 10.1023/B:ASEB.0000026619.95734.d5 15129038

[29] Ashcraft AM Murray PJ. Talking to parents about adolescent sexuality. Pediatr Clin North Am. (2017) 64:305–20. doi: 10.1016/j.pcl.2016.11.002 28292447 PMC5517036

[30] Whitaker DJ Miller KS May DC Levin ML. Teenage partners' communication about sexual risk and condom use: the importance of parent-teenager discussions. Fam Plann Perspect. (1999) 31:117–21. doi: 10.2307/2991693 10379427

[31] Gutu B Mahimbo A Percival N Demant D. Effect of parent-based sexual health education on parent–adolescent communication and adolescent sexual behavior: a systematic review and meta-analysis. Perspect Sex Reprod Health. (2025) 57:374–422. doi: 10.1111/psrh.70029 40762112 PMC12421083

[32] Albert B. With One Voice: America's Adults And Teens Sound Off About Teen Pregnancy. Washington, DC. (2012).

[33] Best C Fortenberry JD. Adolescent Sexuality and Sexual Behavior. In:O'Donohue WT Benuto LT Woodward Tolle L, editors. Handbook of Adolescent Health Psychology. New York, NY: Springer New York (2013). p. 271–91.

[34] Usonwu I Ahmad R Curtis-Tyler K. Parent–adolescent communication on adolescent sexual and reproductive health in sub-Saharan Africa: a qualitative review and thematic synthesis. Reprod Health. (2021) 18:202. doi: 10.1186/s12978-021-01246-0 34629082 PMC8504018

[35] Flores D Barroso J. 21st century parent–child sex communication in the United States: a process review. J Sex Research. (2017) 54:532–48. doi: 10.1080/00224499.2016.1267693 28059568 PMC5808426

[36] Grey EB Atkinson L Chater A Gahagan A Tran A Gillison FB . Systematic review of the evidence on the effect of parental communication about health and health behaviours on children's health and wellbeing. Prev Med. (2022) 159:107043. doi: 10.1016/j.ypmed.2022.107043 35405179

[37] Widman L Evans R Javidi H Choukas-Bradley S. Assessment of parent-based interventions for adolescent sexual health: a systematic review and meta-analysis. JAMA Pediatr. (2019) 173:866–77. doi: 10.1001/jamapediatrics.2019.2324 31355860 PMC6664375

[38] Antwi MA Dogtir JD Islam KAM Alcena-Stiner DC. Adolescent condom negotiation: a concept analysis and conceptual framework. Front Reprod Health. (2026) 8:1764331. doi: 10.3389/frph.2026.1764331 41696455 PMC12894234

[39] Adamek K Meckstroth A Inanc H Ochoa L O'Neil S McDonald K . Conceptual models to depict the factors that influence the avoidance and cessation of sexual risk behaviors among youth. Mathematica Policy Res Rep. (2020):1–18.

[40] Abiodun O Sodeinde K Jagun O Ladele A Adepoju A Ohiaogu F . Influence of perception of family support and functioning on adolescent high-risk sexual behavior. Am J Trop Med Hyg. (2020) 104:1153–63. doi: 10.4269/ajtmh.20-0732 33289467 PMC7941804

[41] Munyai HS Makhado L Ramathuba DU Lebese RT. Challenges regarding sexual health communication with secondary school learners in Limpopo Province: parents views. Curationis. (2023) 46:e1–9. doi: 10.4102/curationis.v46i1.2321 37132564 PMC10157411

[42] Singh DR Shrestha S Karki K Sunuwar DR Khadka DB Maharjan D . Parental knowledge and communication with their adolescent on sexual and reproductive health issues in Nepal. PLoS One. (2023) 18:e0289116. doi: 10.1371/journal.pone.0289116 37490487 PMC10368235

[43] Punjani N Scott SD Hussain A Lu T Bandali F McDonald S . A scoping review of parent-based barriers to parent–child communication about sexuality. Sex Health. (2025) 22:SH25076. doi: 10.1071/SH25076 40977215

[44] Jemmott JB Jemmott LS Braverman PK Fong GT. HIV/STD risk reduction interventions for African American and Latino adolescent girls at an adolescent medicine clinic: a randomized controlled trial. Arch Pediatr Adolesc Med. (2005) 159:440–9. doi: 10.1001/archpedi.159.5.440 15867118

[45] Greenberg JS Bruess CE Oswalt SB. Exploring The Dimensions Of Human Sexuality. 6th ed Burlington (MA): Jones & Bartlett Learning. (2017).

[46] Scull TM Malik C Keefe E. Determining the feasibility of an online, media mediation program for parents to improve parent-child sexual health communication. J Media Lit Educ. (2020) 12:13–25. doi: 10.23860/JMLE-2020-12-1-2 40718574 PMC12291168

[47] New York State Department of Health. Sexually Transmitted Infections Surveillance Report. Albany (NY): New York State Department of Health; 2022.

[48] New York State Department of Health AIDS Institute. Health Advisory: Increased Number Of Hiv Diagnoses In Erie And Monroe Counties. Albany (NY): New York State Department of Health; 2021. p. 5.

[49] Rural Center Council. 2025 childhood poverty report. 2025.

[50] Ruiz-de-Larrinaga M Gutiérrez-García A Potenza MN Demetrovics Z Bothe B McCurdy LY . Comprehensive Sexuality Education: a Qualitative Study on the Perspectives of Teachers, Parents and Adolescents. Sexuality Research and Social Policy. 2026.

[51] Collen LY Green G Umar E Kuchawo MGK Muheriwa-Matemba SR. Building on tradition: using the ADAPT-ITT model to develop a sexual health intervention from initiation rites of passage for rural young women in Southern Malawi. (2296–2565 (Electronic)). 42088227 10.3389/fpubh.2026.1761039 PMC13136122

[52] American Psychological Association. Artificial Intelligence and Adolescent Well-being: An APA Health Advisory. Washington, DC; 2025.

[53] Riehm KE Feder KA Tormohlen KN Crum RM Young AS Green KM . Associations Between Time Spent Using Social Media and Internalizing and Externalizing Problems Among US Youth. JAMA Psychiatry. (2019) 76:1266–73. doi: 10.1001/jamapsychiatry.2019.2325 31509167 PMC6739732

[54] Chen Z Liao X Yang J Tian Y Peng K Liu X . Association of screen-based activities and risk of self-harm and suicidal behaviors among young people: a systematic review and meta-analysis of longitudinal studies. Psychiatry Res. (2024) 338:115991. doi: 10.1016/j.psychres.2024.115991 38833936

[55] Cabezas-Klinger H Fernandez-Daza FF Mina-Paz Y. Associations between social media use and mental disorders in adolescents and young adults: a systematic review and meta-analysis of recent evidence. Behav Sci. (2025) 15. doi: 10.3390/bs15111450 41301253 PMC12649677

[56] Patchin JW. Hinduja S. Sextortion among adolescents: results from a national survey of US youth. Sex Abuse. (2020) 32:30–54. doi: 10.1177/1079063218800469 30264657

[57] The US. Surgeon General's Advisory. Social Media and Youth Mental Health Washington, DC: US Department of Health and Human Services. (2023). 37721985

[58] Campbell LO Babb K Lambie GW Hayes BG. An examination of generative AI response to suicide inquires: content analysis. JMIR Ment Health. (2025) 12:e73623. doi: 10.2196/73623 40811811 PMC12371289

[59] Grundmeier RW Fiks AG Jenssen BP Proctor SN Ferro DF Johnson KB . Generative artificial intelligence: implications for families and pediatricians. Pediatrics. (2026). doi: 10.1542/peds.2025-074912 41775335 PMC13400086

[60] Hoffman K. Florida mother files lawsuit against AI company over teen son's death: “Addictive and manipulative”: CBS News; 2024. Available online at: https://www.cbsnews.com/news/florida-mother-lawsuit-character-ai-sons-death/.

[61] Kågesten A van Reeuwijk M. Healthy sexuality development in adolescence: proposing a competency-based framework to inform programmes and research. Sex Reprod Health Matters. (2021) 29:104–20. doi: 10.1080/26410397.2021.1996116 34937528 PMC8725766

[62] Fairbanks M Lague I. Sex education and HIV education. New York (NY): Guttmacher Institute. (2025).

[63] Nelson KM Gray A Haiken S Underhill K. The state of sexual education: state laws and regulations mandating sexual education in the United States. Am J Public Health. (2025) 115:1723–33. doi: 10.2105/AJPH.2025.308199 40839855 PMC12424484

[64] Panisch LS Faulkner M Fernandez SB Fava NM. Exploring how trauma is addressed in sexual education interventions for youth: a scoping review. Health Educ Behav. (2020) 47:880–93. doi: 10.1177/1090198120954398 32900237

[65] Broussard DL Eitmann LP Shervington DO. Sex education through a trauma-informed lens: do parents who see trauma as a problem for youth support trauma-informed sex education? Am J Sex Educ. (2019) 14:233–57. doi: 10.1080/15546128.2019.1566948

[66] Wesche R Toman M Allen KR Gupta S Grafsky EL. Beyond “the talk”: parent-adolescent sexual socialization in a culture that silences discourse about adolescent sexuality. Fam Relat. (2025) 74:3077–103. doi: 10.1111/fare.70041

[67] Tabana H Satumba T Cooper D. Lembani M. “I tried to commit suicide”: understanding the intersections between mental health, HIV and teenage pregnancy. PLoS ONE. (2026) 21:e0323030. doi: 10.1371/journal.pone.0323030 41490070 PMC12768238

[68] Bodner S. Developing a Shelter-Based Prenatal Syphilis Prevention Program for Pregnant Individuals Experiencing Housing Instability in Allegheny County. 2026.

[69] Tebb KP Brindis CD editors. Understanding the psychological impacts of teenage pregnancy through a socio-ecological framework and life course approach. In: Thieme Medical Publishers, Inc.; 2022. 34991169 10.1055/s-0041-1741518

[70] Baney L Greene A Sherwood-Laughlin C Beckmeyer J Crawford BL Jackson F . ”It was just really hard to be pregnant in a smaller town …": pregnant and parenting teenagers' perspectives of social support in their rural communities. Int J Environ Res Public Health. (2022) 19:16906. doi: 10.3390/ijerph192416906 36554786 PMC9778672

[71] Padilla-Walker LM. Longitudinal change in parent-adolescent communication about sexuality. J Adolesc Health. (2018) 63:753–8. doi: 10.1016/j.jadohealth.2018.06.031 30279105

[72] Collins RL Strasburger VC Brown JD Donnerstein E Lenhart A Ward LM. Sexual media and childhood well-being and health. Pediatrics. (2017) 140:S162–6. doi: 10.1542/peds.2016-1758X 29093054

[73] Kuborn S Markham M Astle S. “I wish they would have a class for parents about talking to their kids about sex”: college women's parent-child sexual communication reflections and desires. Sex Res Soc Policy. (2023) 20:230–41. doi: 10.1007/s13178-022-00723-w

[74] Guse K Levine D Martins S Lira A Gaarde J Westmorland W . Interventions using new digital media to improve adolescent sexual health: a systematic review. J Adolesc Health. (2012) 51:535–43. doi: 10.1016/j.jadohealth.2012.03.014 23174462

[75] Landry M Turner M Vyas A Wood S. Social media and sexual behavior among adolescents: is there a link? JMIR Public Health Surveill. (2017) 3:e28. doi: 10.2196/publichealth.7149 28526670 PMC5457530

